# Development and evaluation of a multiplex loop-mediated isothermal amplification (LAMP) assay for differentiation of *Mycobacterium tuberculosis* and non-tuberculosis mycobacterium in clinical samples

**DOI:** 10.1371/journal.pone.0244753

**Published:** 2021-01-06

**Authors:** Jeeyong Kim, Borae G. Park, Da Hye Lim, Woong Sik Jang, Jeonghun Nam, Do-CiC Mihn, Chae Seung Lim

**Affiliations:** 1 Department of Laboratory Medicine, Korea University Guro Hospital, Seoul, Republic of Korea; 2 Department of Diagnostic Immunology, Seegene Medical Foundation, Seoul, Republic of Korea; Rutgers Biomedical and Health Sciences, UNITED STATES

## Abstract

**Introduction:**

The rapid and accurate diagnosis of tuberculosis (TB) is important to reduce morbidity and mortality rates and risk of transmission. Therefore, molecular detection methods such as a real-time PCR–based assay for *Mycobacterium tuberculosis* (MTB) have been commonly used for diagnosis of TB. Loop-mediated isothermal amplification (LAMP) assay was believed to be a simple, quick, and cost-effective isothermal nucleic acid amplification diagnostic test for infectious diseases. In this study, we designed an in-house multiplex LAMP assay for the differential detection of MTB and non-tuberculosis mycobacterium (NTM), and evaluated the assay using clinical samples.

**Material and methods:**

For the multiplex LAMP assay, two sets of specific primers were designed: the first one was specific for *IS6110* genes of MTB, and the second one was universal for *rpoB* genes of mycobacterium species including NTM. MTB was confirmed with a positive reaction with both primer sets, and NTM was identified with a positive reaction by only the second primer set without a MTB-specific reaction. Total 333 clinical samples were analyzed to evaluate the multiplex LAMP assay. Clinical samples were composed of 195 positive samples (72 MTB and 123NTM) and 138 negative samples. All samples were confirmed positivity or negativity by real-time PCR for MTB and NTM. Analytical sensitivity and specificity were evaluated for the multiplex LAMP assay in comparison with acid fast bacilli staining and the culture method.

**Results:**

Of 123 NTM samples, 121 were identified as NTM and 72/72 MTB were identified as MTB by the multiplex LAMP assay. False negative reactions were seen only in two NTM positive samples with co-infection of *Candida* spp. All 138 negative samples were identified as negative for MTB and NTM. Analytical sensitivity of the multiplex LAMP assay was 100% (72/72) for MTB, and 98.4% (121/123) for NTM. And the specificity of assay was 100% (138/138) for all.

**Conclusions:**

Our newly designed multiplex LAMP assay for MTB and NTM showed relatively good sensitivity in comparison with previously published data to detect isolated MTB. This multiplex LAMP assay is expected to become a useful tool for detecting and differentiating MTB from NTM rapidly at an acceptable sensitivity.

## Introduction

Tuberculosis (TB) has been a leading cause of death especially for low and middle income countries [1]. The rapid and accurate diagnosis of *Mycobacterium tuberculosis* (MTB) infection is important to reduce morbidity and mortality rates and risk of transmission. According to the recent guideline, all of acid-fast bacilli (AFB) smear microscopy, liquid and solid culture for MTB, and nucleic acid amplification tests (NAAT) were recommended for initial clinical sample to diagnose infection of MTB [2]. Among them, NAAT is the most rapid method to detect MTB and the positive NAAT can be used as a presumptive evidence of MTB infection [2]. Therefore, the molecular detection of MTB has been commonly used and the real-time PCR based molecular detection considered as a highly sensitive method for detection [3].

Loop-mediated isothermal amplification (LAMP) assay is an emerging technique which is simple, quick, and cost-effective isothermal nucleic acid amplification diagnostic test for infectious diseases and recommended as diagnosis of pulmonary MTB infection [4]. LAMP is a manual assay that requires about an hour to perform and can be read under visual display with nucleic acid staining (e.g., SYBR Green). Although the sensitivity and specificity of MTB-LAMP is applicable for routine test, but the visual display detection could be less sensitive than real-time PCR based detection [4]. Previously, probe based detection system for LAMP assay could improve the clinical sensitivity and specificities [5].

Recently, the prevalence of non-tuberculosis mycobacterium (NTM) infections are increasing worldwide as well as in South Korea [6, 7]. Therefore, recent real-time PCR based NAATs were developed to detect both MTB and NTM [8, 9]. Until now most of LAMP assay for MTB has been designed only for single detection of MTB or NTM [4]. Therefore, in this study, we designed an in-house multiplex LAMP assay applying with fluorescence-based real-time detection to differentiate MTB and NTM, and we evaluated the novel multiplex LAMP assay comparing with commercially available MTB/NTM real-time PCR kit using clinical samples.

## Material and methods

### Samples preparation and real-time PCR

Total 333 clinical samples from 313 individuals were collected between July 2017 and September 2018. All samples were pre-treated by NaOH and DNA extraction was performed. Sample preparation and DNA extraction was followed by manufacturer’s instruction of Genedia MTB/NTM detection kit (GENEDIA MTB/NTM, Green Cross Medical Science Corp., Chungbuk, Korea) for real-time PCR. The Genedia assay is a real-time PCR method targeting IS6110 and internal transcribed spacer (ITS) region for MTB complex and the rpoB gene for non-tuberculous mycobacterium (NTM). For the assay, 100uL of decontaminated specimens were suspended in 100uL of DNA extraction buffer, and the mixtures were boiled in 10 minutes. After centrifugation for 3 min at 13,000 rpm, the supernatants were used for Genedia MTB/NTM assays. Real time PCR was performed by using the GENEDIA MTB/NTM real-time PCR Kit and ABI 7500 real-time PCR system (Applied Biosystems, Foster City, CA, USA). According to the manufacturer’s guideline, cycle threshold (Ct) less than 40 for IS6110 and 35 for rpoB was interpreted as positive reaction. Any of single positive reaction in IS6110 and *rpoB* is defined as MTB and NTM, respectively. In case of double positive reaction in IS6110 and *rpoB*, if the Ct value of IS6110 lower than that of *rpoB* were as defined as MTB, and that of *rpoB* is lower than IS6110 is defined as double positivity of NTM and MTB based on the manufacturer’s recommendations.

### Loop mediated isothermal amplification (LAMP)

LAMP primers were designed to target IS6110 region specific for *MTB* complex, *rpoB* region specific for both MTB and NTM, and glyceraldehyde-3-phosphate dehydrogenase (GAPDH) gene for internal control (Table 1). The LAMP assay was performed using LAMP reagent kit (M-monitor, Daegue, Korea). Total 25.5 μL reaction mixture containing 2.5 μL of DNA template, 0.5 μL of IS6110 specific primer set, 1.2 uL of rpoB specific primer set, and 0.5 μL of primer set targeting GAPDH internal control gene. The reaction tubes were loaded into a thermocycler (CFX-96, Bio-Rad, Korea) at 65°C for 60 minutes and 80°C for 5 minutes. Any of positive fluorescence result was detected with Ct value and the positive IS6110 specific reaction was confirmed as MTB positivity regardless of rpoB positivity, and positive reaction of rpoB region with negative IS6110 specific reaction was confirmed as NTM positivity. Single positive reaction of internal control (GAPDH) was judged as negative for MTB and NTM in LAMP assay.

**Table 1:** Nucleotide sequences of the LAMP primers for *Mycobacterium tuberculosis*, non-tuberculosis mycobacterium, and internal control sequence of GAPDH gene.

LAMP PCR primers (5’ to 3’) targeting IS6110 region of *Mycobacterium tuberculosis*	
MTB_F3	GGTCGGAAGCTCCTATGACA
MTB_B3	TAGGCAGCCTCGAGTTCG
MTB_FIP (F1c-F2)	AGGGCTTGCCGGGTTTGATC-ATGCACTAGCCGAGACGA
MTB_BIP (B1c-B2)	CGGTCCATCGAGGATGTCGAGT-CGCCGCAGTACTGGTAGA
MTB_FLP	CTCGGTCTTGTATAGGCCGT
MTB_BLP	ACCGCGCGCTGGGTCGA
MTB_BLP_probe_Cy5	GTCAGTGCAGGCTCCCGTGTTAGGACGAGGGTAGGACCGCGCGCTGGGTCGA
LAMP PCR primers (5’ to 3’) targeting *rpoB* gene region of Mycobacterium (both MTB and NTM)	
NTM_F3	CCGGTCACCGTGCTG
NTM_B3	GTARCGCTTCTCCTTGAAGAAC
NTM_FIP (F1c-F2)	TGTTGTCCTTCTCCAGIGTIIGCTGGACCAICGAGCA
NTM_BIP (B1c-B2)	TGGACATCTACCGCAAGCTGCGTTYTCCARCAGGGTCTGC
NTM_FLP	CGGAGAAICCGAAICGCTC
NTM_BLP	CCGCCGACCAAAGAGTCAGC
NTM_FLP_probe TEX	TEXASRED-CCGCCGACCAAAGAGTCAGC-BHQ1
Internal control primers (5’ to 3’) targeting GAPDH gene	
IC_F3	TCC GCA CAT CCC GGT ATG
IC_B3	TCT GCA GAT AGG AAG GGC TT
IC_FIP	TGG GGG CAT TGA AGG GGT GAT GAG GGT TCT TTG TGC TGA G
IC_BIP	CTG TGG CAT CCC TGG GAC TGG TGA GGA ACT GGG AGA TCC A
IC_FLP	ATG GGC CTT TCT CCC CTG C
IC_BLP	GGG GAA GGT TGA GCC TTT ACT
IC_BLP probe_HEX	CGGGCCCGTACAAAGGGAACACCCACACTCCG GGG GAA GGT TGA GCC TTT ACT

Abbreviations: LAMP, loop mediated isothermal amplification polymerase chain reaction; MTB, *Mycobacterium tuberculosis*; NTM, Non-tuberculosis Mycobacterium; GAPDH, Glyceraldehyde-3-phosphate denydrogenase.

### Smear and culture

AFB smear and culture were performed for all 333 samples. AFB smears were performed using auramine-rhodamine fluorescent staining. And the sediment of NaOH pre-treated sample was cultivated in VersaTREK system (Trek Diagnostic System, ThermoFisher, Cleveland, OH, USA) for 6 weeks as well as on the 3% Ogawa solid media for 8 weeks in 5–10% CO2 incubators. Differentiation of MTB in positive cultures was performed using the SD Bioline MTB Ag MPT64 RAPID kit (Standard Diagnostics, Seoul, Korea). Positive culture sample with negative MTB Ag results performed real-time PCR to differentiate MTB and NTM.

### Assay sensitivity analysis with limit of detection

To determine limit of detection, a 10-fold serial dilution series of the IS6110 plasmid (1x10^8^ copies/uL) was used for current multiplex LAMP assay using fluorescence probe (Cy5, TEX, and HEX, Table 1). Dye based detection was also performed in parallel by using WarmStart^®^ Colorimetric LAMP 2X Master Mix DNA & RNA (New England Biolabs Inc, MA, USA).

### Statistical analysis and ethic statement

Statistical analyses were carried out using the MedCalc Statistical Software version 19.0.4 (MedCalc Software bvba, Ostend, Belgium; https://www.medcalc.org; 2019). Test agreement was presented as percent agreement and kappa coefficient. This study was approved by the Institutional Review Board (IRB) of Korea University Guro Hospital (2019GR0055). The need for consent was waived by IRB approval.

## Result

### Real-time PCR, smear and culture positivity for mycobacterium

**Fig 1:**
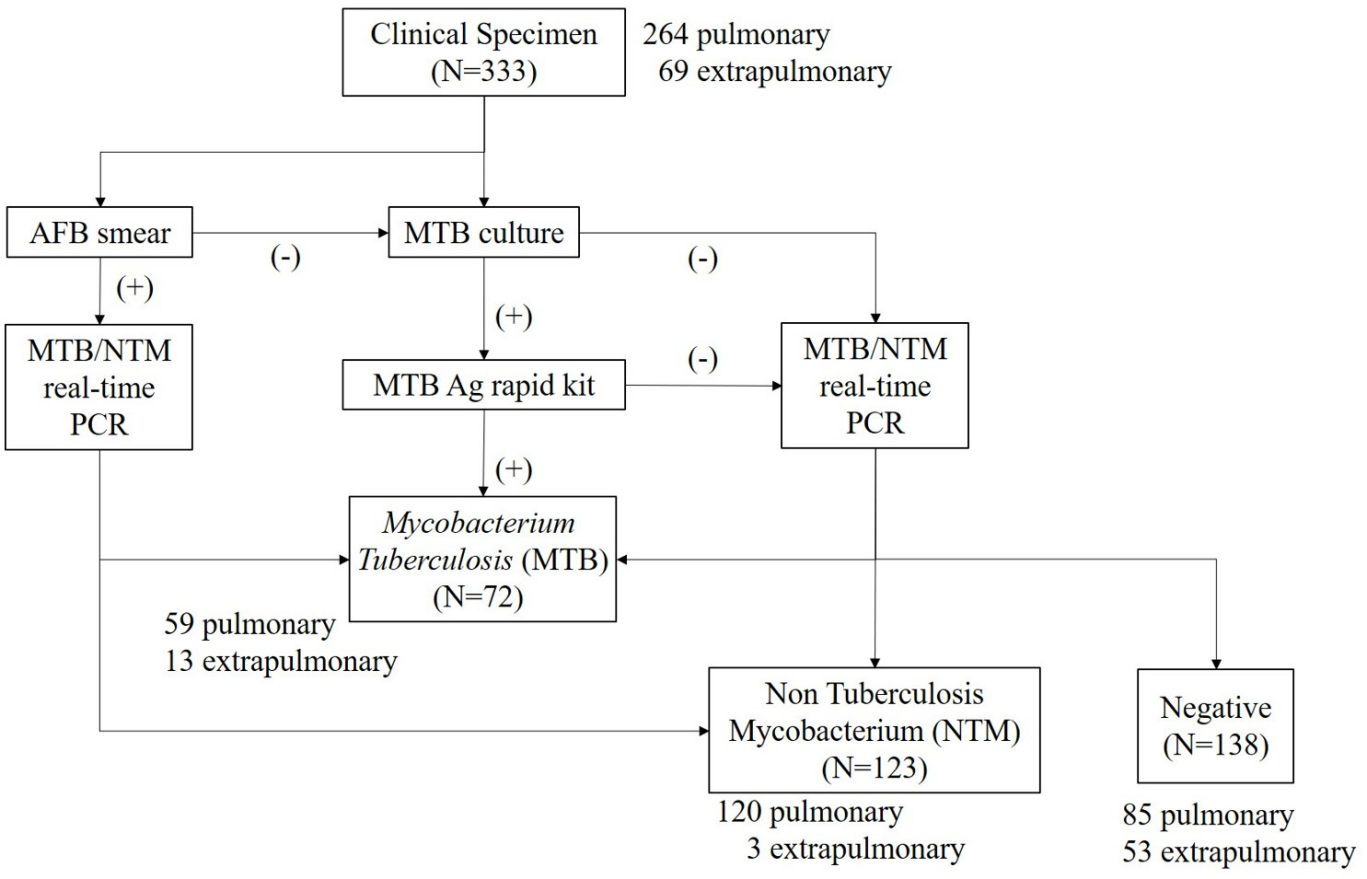
Study algorithm. Algorithms to classify the study samples. All negative samples (N = 138) were negative in AFB (acid-fast bacilli) smear (auramine-rhodamine fluorescent staining), real-time PCR and no growth in MTB culture. MTB and NTM samples were classified according to the real-time PCR identification.

Of the 333 samples, 72 (21.6%) were identified as MTB in Real-time PCR regardless of culture positivity, 138 (41.4%) were negative in real-time PCR and no growth in MTB culture, and 123 (36.9%) were identified as NTM in both real-time PCR and culture (Fig 1). Culture positive MTB was 73.6% (53/72) and AFB smear positive MTB was 34.7% (25/72) of MTB PCR positive samples (Table 2). Total 26.0% (32/123) of NTM culture positive samples showed AFB smear positivity. Most of specimens were composed of sputum and bronchial washing and positive PCR rate in these specimens were 78.9% (123/156) and 51.4% (55/107), respectively (Table 3). Of the 333 samples, 264 samples were pulmonary specimens such as bronchial washing and sputum. Both positive and negative samples were composed of the pulmonary and extrapulmonary specimens.

**Table 2:** Comparison of real time-PCR positive rates according to AFB stain smear grade and culture results (N = 333).

AFB stain	Mycobacterial culture	MTB-PCR positive	NTM-PCR positive	Real-time PCR Negative
Negative	No growth	19 (5)*		138 (53)
	*Mycobacterium tuberculosis*	28 (6)		
	Non-tuberculosis Mycobacterium		91 (2)	
1+	*Mycobacterium tuberculosis*	16 (2)		
	Non-tuberculosis Mycobacterium		9 (1)	
2+	*Mycobacterium tuberculosis*	4		
	Non-tuberculosis Mycobacterium		13	
3+	*Mycobacterium tuberculosis*	1		
	Non-tuberculosis Mycobacterium		3	
4+	*Mycobacterium tuberculosis*	4		
	Non-tuberculosis Mycobacterium		7	
Total		72 (13)	123 (3)	138 (53)

*Data for extrapulmonary specimens were indicated in the parentheses.

Abbreviations: AFB, acid fast bacilli; MTB, *Mycobacterium tuberculosis*; NTM, Non-tuberculosis Mycobacterium.

**Table 3:** Real-time PCR result distribution according to the specimen (N = 333).

Specimen		Real-time MTB PCR positive*						Real-time PCR NTM positive						Negative**	Total
AFB stain		Neg	1+	2+	3+	4+	total	Neg	1+	2+	3+	4+	total	Neg	
Pulmonary	Bronchial washing	17(16)†	9(8)	2		2	30(28)	10	5(4)	7(6)		3	25(23)	52	107(103)
	Sputum	19(18)	5	2(1)	1	2	29(27)	79(73)	3	6(5)	3	4(3)	95(87)	33(32)	157(146)
Total		36(34)	14(13)	4(3)	1	4	59(55)	89(83)	8(7)	13(11)	3	7(6)	120(110)	85(84)	264(249)
Extrapulmonary	Cerebrospinal fluid						0						0	14(13)	14(13)
	Joint or bone aspiration	1					1	1					1	10	12
	Pleural fluid or ascites	1					1		1				1	23(22)	25(24)
	Lymph node or tissue biopsy	6(5)					6(5)						0	1	7(6)
	Pus	3(2)	2(1)				5(3)						0	4	9(7)
	Urine						0						0	1	1
	Stool						0	1					1	0	1
Total		11(9)	2(1)	0	0	0	13(10)	2	1	0	0	0	3	53(51)	69(64)

*Total 19 of TB PCR positive samples showed no growth in TB culture results.

** All of these samples showed negative for AFB stain and no growth for TB culture results.

^†^In case of duplicate collection, the number of patients were indicated in parentheses.

Abbreviations: Neg, negative; MTB, *Mycobacterium tuberculosis*; AFB, acid fast bacilli; NTM, Non-tuberculosis Mycobacterium.

#### Analytical sensitivity and specificity of multiplex LAMP assay comparing with real-time PCR

In comparison with real-time PCR, overall sensitivity of LAMP assay was 98.97% (193/195), specificity of LAMP assay was 100% (138/138), and weighted Kappa score was 0.9925 in agreement analysis results between LAMP and real-time PCR (Table 4). For pulmonary specimens, analytical sensitivity was 98.88% (177/179) and specificity was 100% (85/85). All MTB positive samples in real-time PCR showed positive reaction in LAMP assay (72/72). However, 2 of NTM positive samples (2/123) in real-time PCR were not amplified in LAMP assay (Table 4).

**Table 4:** Overall analytical sensitivity and specificity and agreement analysis of LAMP assay in comparison with real-time PCR assay (N = 333).

	Real-time PCR	MTB LAMP positive	NTM LAMP positive	LAMP negative	Total
Comparison	MTB	72	0	0	72 (21.6%)
	NTM		121	2	123 (36.9%)
	Negative			138	138 (41.4%)
	Total	72 (21.6%)	121 (36.3%)	140 (42.0%)	
Comparison for pulmonary specimens (N = 264)	MTB	59	0	0	59 (22.3%)
	NTM		118	2	120 (45.5%)
	Negative			85	85 (32.2%)
	Total	59 (22.3%)	118 (44.7%)	87 (33.0%)	
Overall strength comparing with real-time PCR for pulmonary specimens (N = 264)	Analytical sensitivity	98.88% (96.02–99.86)			
	Analytical specificity	100% (95.75–100)			
	NPV	97.70% (91.46–99.41)			
	PPV	100% (NA)			
Overall strength comparing with real-time PCR (N = 333)	Analytical sensitivity	98.97% (96.34–99.88)			
	Analytical specificity	100% (97.36–100)			
	NPV	98.57% (94.56–99.64)			
	PPV	100% (NA)			
Agreement analysis*	Weighted Kappa (95% CI)	0.9925 (0.9821–1.0000)			
	Standard error	0.0053			
	Indicators	Overall (95% CI)			

*Kappa agreement analysis applied.

Abbreviations: LAMP, loop-mediated isothermal amplification assay; MTB, *Mycobacterium tuberculosis*; NTM, Non-tuberculosis Mycobacterium; CI, confidence interval, NPV, negative predictive value, PPV, positive predictive value.

**Fig 2:**
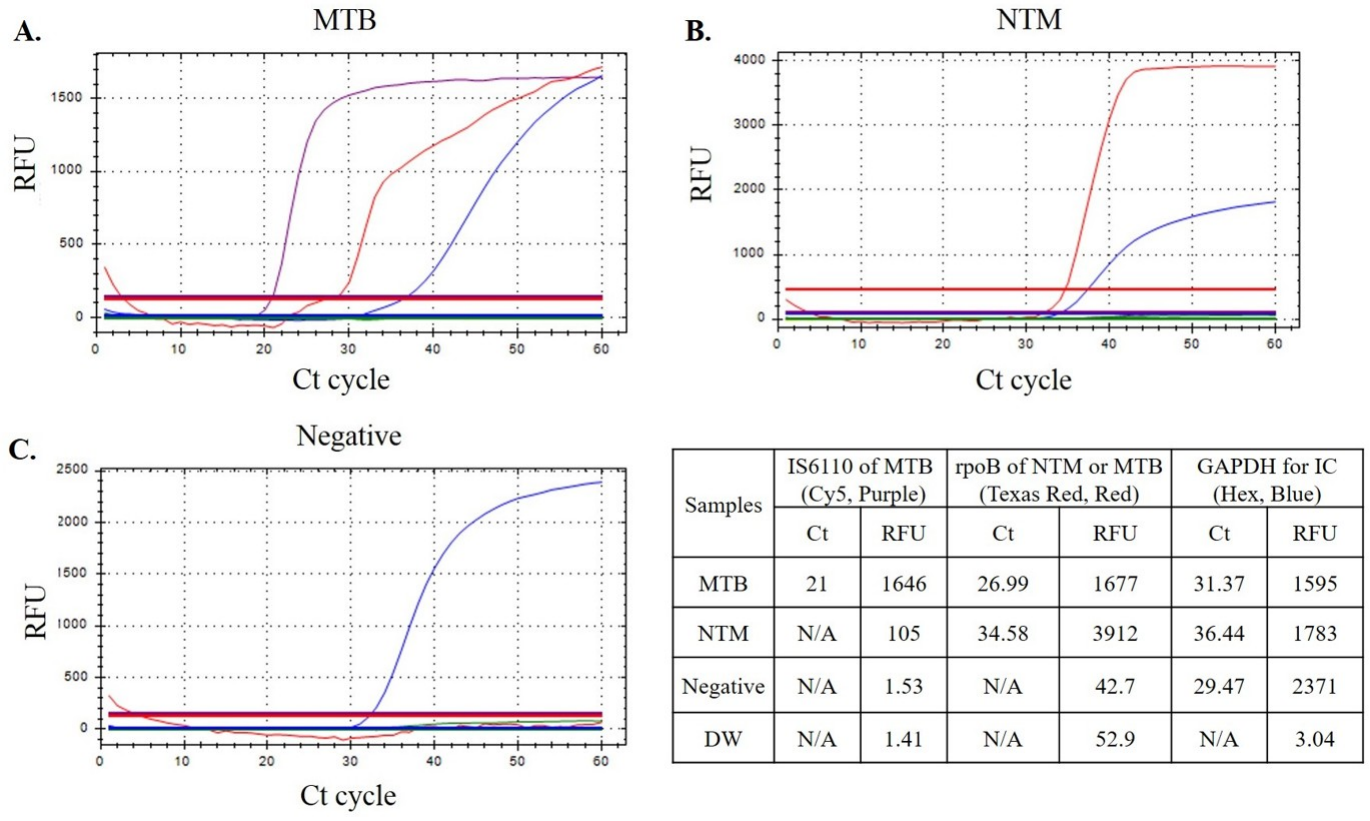
Example of result. Fluorescence detection of MTB/NTM-LAMP results for positivity of MTB (purple, Ct value for MTB: 21.0, A), B. NTM (red, Ct value for NTM: 34.58, B), and Negative (C), respectively. Abbreviations: LAMP; loop mediated isothermal amplification, MTB; *Mycobacterium tuberculosis*, NTM; non-tuberculosis mycobacterium, GAPDH; human genomic DNA for Glyceraldehyde 3-phosphate dehydrogenase, IC; internal control, RFU; Relative fluorescence units, Ct; Cycle thresholds.

For detection of MTB both IS6110 and *rpoB* genes were amplified. And Ct value of IS6110 for MTB in LAMP assay was shorter than in real-time PCR (21.31±5.04 vs 31.00±5.40). Also, for all MTB positive reaction Ct value of IS6110 was shorter than those of *rpoB* (with 9.40±4.00 of delta Ct value, Table 5, Fig 2). LAMP assay is not a real-time PCR, therefore, there was no cut-off for positive Ct value of LAMP assay. However, except one sample (54.41 for IS6110 and 56.99 for *rpoB* Ct value, sputum, no growth in culture), all MTB samples were detected within 35 Ct values in LAMP assay.

**Table 5:** Cycles of threshold values of LAMP and real-time PCR assay according to the positive results of organisms.

	LAMP-MTB			Real-time PCR Ct value of IS6110 for MTB (<40.00)	LAMP-NTM* Ct value of rpoB for NTM	Real-time PCR Ct value of rpoB for NTM (<35.00)
	Ct value of IS6110 for MTB	Ct value of rpoB for MTB	Delta Ct value of rpoB—IS6110			
Mean	21.31	30.71	9.40	31.00	26.58	25.28
SD	5.04	6.04	4.00	5.40	6.44	3.36
Minimum	14.39	20.73	1.78	18.30	18.13	20.00
Maximum	54.41	56.99	19.60	39.20	54.48	34.00
Total Number of reaction	72	72	72	72	121	123

*Ct values of IS6110 gene amplification were all negative in NTM positive samples.

Abbreviations: LAMP, loop-mediated isothermal amplification assay; MTB, *Mycobacterium tuberculosis*; NTM, Non-tuberculosis Mycobacterium; Ct value, Cycles of threshold.

For detection of NTM, IS6110 amplification was all negative and only the Ct value of *rpoB* genes was detected. And Ct values were similar between LAMP and real-time PCR (26.58±6.44 and 25.28±3.36).

### Detection of non-tuberculosis mycobacterium in multiplex LAMP assay

Total 76 sample was identified as subspecies of NTM by the culture and identification, 9 kinds of various NTMs were included in this study (Table 6). Mostly identified NTMs were composed of *M*. *intracellulare (38*.*2%)*, *M*. *avium (35*.*5%)*, and *M*. *abscessus (11*.*8%)*, in order. Total 2 of NTM positive specimen, which were co-infected with *Candida* spp. (*C*. *albicans* and *C*. *glabrata*, respectively) were not amplified in the multiplex LAMP assay, and 5 NTMs were detected over 40 of Ct values (Table 7). Among these 7 NTMs, 5 were negative in AFB stain but 2 of them showed 2+ and 4+ positivity in AFB stain. Total 12 NTMs were co-cultured with *Candida* spp. in this study (Table 8) and 3 of candida co-cultured samples showed negative or delayed Ct values in LAMP assay.

**Table 6:** Identification results for non-tuberculosis mycobacterium (N = 76).

Specimen	Number	AFB positive	AFB negative
*M*. *intracellulare*	29	11	18
*M*. *avium*	27	5	22
*M*. *abscessus*	9	4	5
*M*. *massiliense*	4	2	2
*M*. *kansasii*	1	0	1
*M*. *marinum*	1	0	1
*M*. *chelonae*	1	0	1
*M*. *gordona*	1	0	1
*M*. *lentiflavum or genavense*	1	0	1
Mixed (*M*. *avium and M*. *intracellulare*)	2	2	0

*M*, *Mycobacterium*; AFB, acid fast bacilli.

**Table 7:** Data on 2 NTM results that were discordant between real-time PCR and LAMP assay and 5 results which were detected as positivity with more than 35 of Ct values in LAMP assay.

Specimen	AFB	Culture	CT value of Real-time PCR	Ct value of LAMP
Sputum	4+	NTM and *Candida albicans*	25.0	Negative
Bronchial washing	Negative	NTM and *Candida glabrata*	29.0	Negative
Sputum	Negative	*M*. *kansasii*	23.6	54.48
Sputum	Negative	NTM	25.4	54.48
Sputum	Negative	NTM	22.1	46.61
Bronchial washing	Negative	NTM with *Candida albicans*	26.4	40.57
Bronchial washing	2+	NTM	25.4	40.02

Abbreviations: NTM, Non-tuberculosis Mycobacterium; LAMP, loop-mediated isothermal amplification assay.

**Table 8:** Characteristics of NTM positive specimens which were co-infected with *Candida* spp. (N = 12).

Microorganisms for mixed infection	Identification	Specimen	AFB	Real-time PCR Ct values for NTM positivity (Ct <35)	LAMP Ct values for NTM positivity
*Candida albicans* (N = 8)	*M*. *intracellulare*	Sputum	1+	32.0	26.96
	*M*. *intracellulare*	Bronchial washing	Negative	30.7	30.62
	*M*. *abscessus*	Sputum	2+	26.6	25.14
	*M*. *avium*	Sputum	3+	33.0	28.93
	NTM	Sputum	4+	25.0	Negative*
	NTM	Sputum	Negative	22.4	23.02
	NTM	Sputum	Negative	25.0	23.98
	NTM	Bronchial washing	Negative	26.4	40.57*
*Candida glabrata* (N = 4)	*M*. *intracellulare*	Sputum	Negative	21.9	23.37
	*M*. *abscessus*	Sputum	2+	25.5	26.69
	NTM	Sputum	Negative	23.5	22.27
	NTM	Bronchial washing	Negative	29.0	Negative*

Abbreviations: NTM, Non-tuberculosis Mycobacterium; LAMP, loop-mediated isothermal amplification assay.

### Limit of detection for *Mycobacterium tuberculosis*

**Fig 3:**
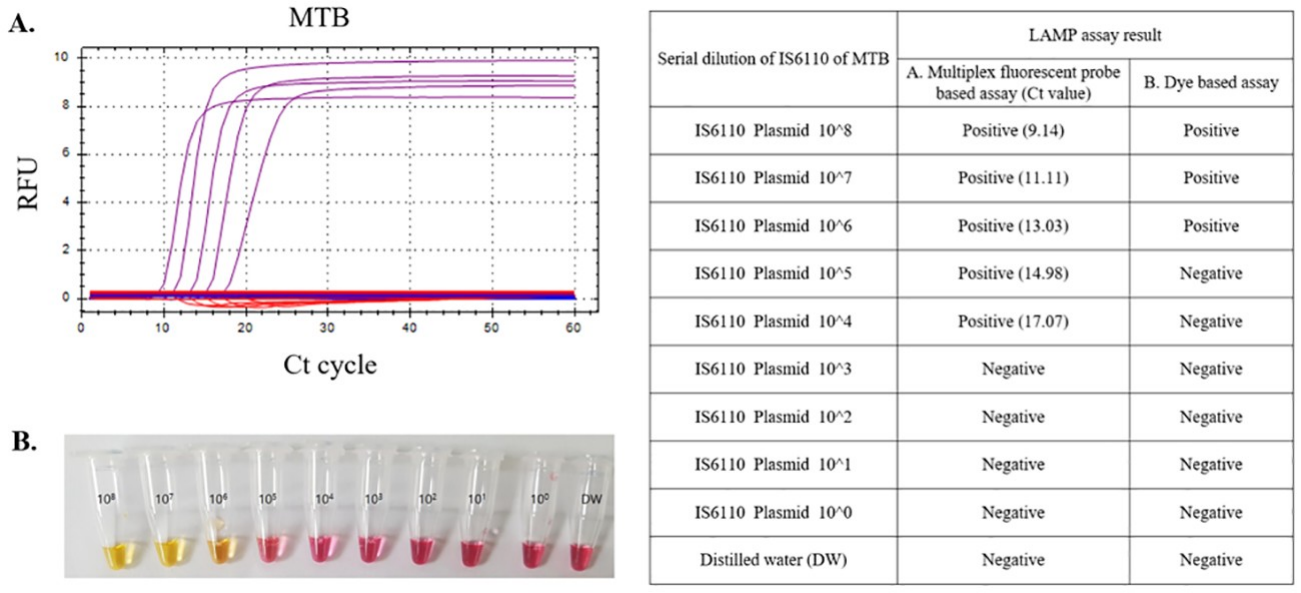
Limit of detection. The limit of detection for *Mycobacterium tuberculosis* between the multiplex LAMP (A) and dye based LAMP assay (B). LAMP; loop mediated isothermal amplification, MTB; *Mycobacterium tuberculosis* Ct; Cycle thresholds.

A ten-fold serial dilution of the IS6110 plasmid template showed that the multiplex LAMP was sensitive to 1x10^4^ copies/uL. In contrast, the dye based detection system could only detect the IS6110 plasmid template down to 1x10^6^ copies/uL level (Fig 3).

## Discussion

Until now numerous LAMP assays were introduced for MTB detection [4]. In previous LAMP study for MTB, pooled sensitivity was 0.93 (0.79–1.00) and pooled specificity was 0.94 (0.48–0.99) for sputum samples in comparison with MTB culture positivity [10]. The other meta-analysis reported overall summarized sensitivity as 89.6% and specificity as 94.0% in sputum samples [11]. In the present study, overall sensitivity of LAMP assay was 98.97% and specificity was 100% in comparison with real-time PCR results. These might mean that sensitivity and specificity of current multiplex LAMP assay was superior to previous reported performance value. Traditionally, amplified MTB-LAMP product alone could be detected under naked eye with SYBR green dye staining. However, probe based detection system for LAMP assay showed better performance value than original LAMP system detection under naked eye [5]. Also, in this study, multiplex LAMP assay with fluorescence-based detection showed better limit of detection for MTB than naked eye detection (Fig 3). Therefore, our results with better sensitivity and specificity of multiplex LAMP system to detect MTB than traditional LAMP systems were consistent with the results of previous report [5].

In addition to better sensitivity and specificity for MTB, this multiplex LAMP system could distinguish MTB from NTM. However, for the NTM results, 2 NTMs were not detected and 5 NTMs were detected over 40 Ct values in multiplex LAMP assay. These findings might be meaning relatively low sensitivity of multiplex LAMP assay for NTM positivity. Total 3 of 12 samples which were co-cultured with *Candida albicans* or *glabrata* were not detected or detected in over 40 of Ct values. Therefore in case of patient with *Candida* co-infection, such as immunocompromised patient, the sensitivity of assay could be less than expected. However, to confirm this kind of interference by *Candida* organisms, further study would be necessary.

In this study, 19 of MTB showed positivity in both real-time PCR and LAMP assay with negative AFB smear and culture. Among them, 17 patients had previously treatment history of tuberculosis. Therefore these positive NAAT results could be for non-viable MTB. However, 2 of them were new onset of active pulmonary tuberculosis in radiological findings. Also, these 2 patients showed culture positivity in additional sampling. Both specimens were sputums, in some cases, NAAT might be able to sensitive detection of pulmonary TB.

Additionally, for the non-pulmonary samples, the sensitivity and specificity was reported as 93.3%, 91.9%, respectively [11]. In current study, the sensitivity was 100% (17/17) and specificity was 100% (53/53) for the non-pulmonary samples. Current multiplex LAMP assay showed good performance for the non-pulmonary samples, however total number of specimen was only 17 samples. Therefore the further study with more samples would be required to clarify the interference of assay.

Usually, NALC-NaOH or NaOH pretreatments were applied for the sample preparation [12]. In this study, NaOH treated DNA was used for the multiplex LAMP assay. Not only for LAMP but also for real-time PCR, NaOH pretreatment was necessary for prevention of interference by normal flora in the specimen. Same NaOH pretreatment procedure works well for both multiplex LAMP and real-time MTB PCR. Therefore the multiplex LAMP assay in this study would not affect on the NALC-NaOH or NaOH pretreatments.

In summarize, LAMP assay with fluorescence-based detection could improve the sensitivity of LAMP assay and make possible of multiplexing detection in LAMP system. Although there were several false negative NTM cases, but our newly designed multiplex LAMP assay for MTB and NTM showed relatively good sensitivity in comparison with previously published data [4]. In conclusion, this multiplex LAMP assay is expected to become a useful tool for detecting and differentiating MTB from NTM rapidly with acceptable sensitivity.

## References

[1] Furin J, Cox H, Pai M. Tuberculosis. The Lancet. 2019;393(10181):1642–56. 10.1016/s0140-6736(19)30308-3 30904262

[2] Lewinsohn DM, Leonard MK, LoBue PA, Cohn DL, Daley CL, Desmond E, et al Official American Thoracic Society/Infectious Diseases Society of America/Centers for Disease Control and Prevention Clinical Practice Guidelines: Diagnosis of Tuberculosis in Adults and Children. Clin Infect Dis. 2017;64(2):e1–e33. 10.1093/cid/ciw694 27932390

[3] Babafemi EO, Cherian BP, Banting L, Mills GA, Ngianga K 2nd. Effectiveness of real-time polymerase chain reaction assay for the detection of Mycobacterium tuberculosis in pathological samples: a systematic review and meta-analysis. Syst Rev. 2017;6(1):215 10.1186/s13643-017-0608-2 29070061 PMC5657121

[4] World Health Organization. The use of loop-mediated isothermal amplification (TB-LAMP) for the diagnosis of pulmonary tuberculosis: policy guidance. Geneva: World Health Organization; 2016 https://apps.who.int/iris/handle/10665/249154 27606385

[5] Gadkar VJ, Goldfarb DM, Gantt S, Tilley PAG. Real-time Detection and Monitoring of Loop Mediated Amplification (LAMP) Reaction Using Self-quenching and De-quenching Fluorogenic Probes. Sci Rep. 2018;8(1):5548 10.1038/s41598-018-23930-1 29615801 PMC5883045

[6] Lee H, Myung W, Koh WJ, Moon SM, Jhun BW. Epidemiology of Nontuberculous Mycobacterial Infection, South Korea, 2007–2016. Emerg Infect Dis. 2019;25(3):569–72. 10.3201/eid2503.181597 30789139 PMC6390769

[7] Prevots DR, Marras TK. Epidemiology of human pulmonary infection with nontuberculous mycobacteria: a review. Clin Chest Med. 2015;36(1):13–34. 10.1016/j.ccm.2014.10.002 25676516 PMC4332564

[8] Jung YJ, Kim JY, Song DJ, Koh WJ, Huh HJ, Ki CS, et al Evaluation of three real-time PCR assays for differential identification of Mycobacterium tuberculosis complex and nontuberculous mycobacteria species in liquid culture media. Diagn Microbiol Infect Dis. 2016;85(2):186–91. 10.1016/j.diagmicrobio.2016.03.014 27105774

[9] Opota O, Mazza-Stalder J, Greub G, Jaton K. The rapid molecular test Xpert MTB/RIF ultra: towards improved tuberculosis diagnosis and rifampicin resistance detection. Clin Microbiol Infect. 2019;25(11):1370–6. 10.1016/j.cmi.2019.03.021 30928564

[10] Yan L, Xiao H, Zhang Q. Systematic review: Comparison of Xpert MTB/RIF, LAMP and SAT methods for the diagnosis of pulmonary tuberculosis. Tuberculosis (Edinb). 2016;96:75–86. 10.1016/j.tube.2015.11.005 26786658

[11] Nagai K, Horita N, Yamamoto M, Tsukahara T, Nagakura H, Tashiro K, et al Diagnostic test accuracy of loop-mediated isothermal amplification assay for Mycobacterium tuberculosis: systematic review and meta-analysis. Sci Rep. 2016;6:39090 10.1038/srep39090 27958360 PMC5153623

[12] Buijtels PC, Petit PL. Comparison of NaOH-N-acetyl cysteine and sulfuric acid decontamination methods for recovery of mycobacteria from clinical specimens. J Microbiol Methods. 2005;62(1):83–8. 10.1016/j.mimet.2005.01.010 15823396

